# Molecular mechanisms and therapeutic implications of the sympathetic nervous system in bone-related disorders: a brain-bone axis perspective

**DOI:** 10.1038/s41413-025-00494-1

**Published:** 2025-12-02

**Authors:** Mingdong Liu, Yaqi Liu, Jiayao Yu, Jiaqi Gong, Chunguang Zhao, Zheng Liu

**Affiliations:** 1 Department of orthopaedics, Affiliated Hospital of Shaoxing University, Shaoxing, Zhejiang China; 2 Department of Nursing, School of Medicine, Shaoxing University, Shaoxing, Zhejiang China; 3 Department of Forensic Toxicology, Shaoxing University Forensic Center, Shaoxing, Zhejiang China; 4 Department of Pharmacology, School of Medicine, Shaoxing University, Shaoxing, Zhejiang China

**Keywords:** Bone, Hypothalamus

## Abstract

The global aging crisis has increased the prevalence of skeletal disorders, necessitating innovative therapeutic strategies. This review employs the brain-bone axis (BBA) framework to examine the role of the sympathetic nervous system (SNS) in bone metabolism. The research systematically elucidates the molecular mechanisms by which the SNS mediates signaling pathways through neurofibers and neurotransmitters, such as norepinephrine, dopamine, neuropeptide Y, and leptin, regulating interactions between bone-related cells to maintain skeletal homeostasis. It also identifies the pathological associations between the dysregulation of these pathways and the progression of bone-related conditions, such as osteoporosis, osteoarthritis, and intervertebral disc degeneration. By integrating current evidence, we identify novel therapeutic targets within the BBA and propose neuro-centric intervention strategies to mitigate skeletal diseases. This review deepens the understanding of neuro-skeletal interactions and lays a foundation for innovative treatments for bone-related pathologies.

## Introduction

The trend toward rapid aging in the global population is projected to result in approximately 2.1 billion individuals aged 60 and above by the mid-21st century, significantly exacerbating the prevalence of age-related skeletal disorders and posing a critical public health challenge worldwide.^1^ Epidemiological studies have demonstrated a strong correlation between skeletal diseases and aging. In 2020, the number of patients with osteoarthritis (OA) was approximately 595 million, with projected increases of 75% and 50% in knee and hand OA cases, respectively, over the next three decades.^2^ The incidence of osteoporotic fractures is expected to rise to 3.3 million cases by 2030, with associated healthcare expenditures reaching an estimated €47.4 billion.^3^ This emerging crisis has prompted a paradigm shift in orthopedic research, notably through the identification of the brain-bone axis (BBA), a bidirectional neuro-skeletal communication network centered on the sympathetic nervous system (SNS).^4^ Recent evidence suggests that dysregulation of the SNS constitutes a pivotal pathogenic mechanism underlying osteoporosis, OA progression, and intervertebral disc degeneration (IVDD). This review systematically describes the sympathetic nerve-mediated mechanisms of bone metabolism within the BBA framework and evaluates the potential therapeutic implications for skeletal diseases.

The BBA research domain has evolved, and the intricate crosstalk between the nervous system and skeletal metabolism has been elucidated.^5^ Traditionally regarded as functionally independent entities, the nervous and skeletal systems are now confirmed to be interconnected through bidirectional communication networks.^6^ As regulators of systemic homeostasis, the central nervous system (CNS) orchestrates physiological interactions among various organs. Skeletal tissue provides mechanical support and plays multifaceted roles, including hematopoiesis, endocrine regulation, and metabolic functions.^7^ This paradigm shift is comprehensively reflected within the conceptual framework of the BBA, which delineates how neural signals modulate osteoclast and osteoblast activity, as well as how osteogenic factors influence neurophysiological processes. Notably, psychological stress, emotional disorders, and neurodegenerative diseases are associated with accelerated bone loss, underscoring the clinical significance of the BBA.^8,9^

The SNS is a principal branch of the autonomic nervous system. It has been established as a critical neurobiological hub regulating bone metabolism.^10^ Its classical function involves mediating the “fight-or-flight” stress response by rapidly mobilizing physiological resources, such as modulating the heart rate, blood pressure, vasomotor tone, metabolic activity, and glandular secretions. Anatomically, the cell bodies of sympathetic neurons innervating bone tissue are located in the lateral horns of thoracolumbar spinal cord segments T1-L2/3. Preganglionic fibers originate from the ventral roots of the spinal nerves. After synapsing within the sympathetic chain ganglia or prevertebral ganglia, postganglionic fibers extensively project to effectors, including skeletal tissue.^11^ Sympathetic fibers are predominantly distributed within the periosteum and the bone marrow (BM) microenvironment, where their neurotransmitter release exhibits specific patterns: preganglionic terminals release acetylcholine (ACh), whereas the majority of postganglionic fibers innervating bone tissue predominantly release norepinephrine (NE).^12,13^ This precisely targeted neural innervation and the release of key neurotransmitters is where the SNS extends beyond its traditional role in stress response, exerting direct regulatory effects on bone metabolic processes.^14^

In the BBA bidirectional communication network, the SNS modulates osteo-neural interactions through neurofibers and their released neurotransmitters, neuropeptides, and regulatory factors, such as leptin. These molecules bind to specific receptors on osteocytes and activate distinct intracellular signaling cascades that finely regulate the activity of osteoblasts and osteoclasts, thereby maintaining skeletal homeostasis.^15,16^ Recent studies have further elucidated the pivotal role of SNS-mediated neuroregulatory mechanisms in the pathogenesis of osteoporosis and OA, highlighting the therapeutic potential of targeting SNS signaling pathways in skeletal disorders.^17,18^

This review systematically examines the mechanistic roles of SNS in osteo-related pathologies, explores its regulatory functions in bone metabolism, and proposes novel neuroregulatory therapeutic strategies. This study advances our understanding of neuroskeletal interactions and identifies innovative treatment approaches for orthopedic diseases by integrating existing evidence.

## Overview of the BBA

### Neuroanatomical basis of the BBA

Research has confirmed that the skeletal system functions not only as the structural support of the human body but also as the largest endocrine organ. Bones can secrete a variety of osteokines, such as fibroblast growth factor 23 (FGF23), prostaglandin E2 (PGE2), transforming growth factor-beta (TGF-β), osteocalcin (OCN), sclerostin (SOST), nuclear factor kappa-Β ligand (RANKL), lipocalin-2 (LCN-2), and osteopontin (OPN), which regulate the functions of peripheral organs.^19^ Among these, OCN, receptor activator of RANKL, LCN-2, and OPN can cross the blood-brain barrier (BBB), exerting direct effects on the nervous system and participating in neuroregulatory processes.^20^ The bidirectional, osteokine-mediated functional interaction between the skeleton and the brain has increasingly become a research focal point. This complex communication network is anatomically supported by bone innervation throughout cortical and trabecular bone, BM, and the periosteum.^21,22^ The homeostasis of bone tissue relies on a sophisticated cellular network comprising mesenchymal stem cells (MSCs), osteoblasts, osteoclasts, chondrocytes, and myeloid cells, which coordinate dynamically through intricate interactions to regulate bone formation, remodeling, and resorption processes.^23,24^

The CNS modulates the bone remodeling process through multiple neural pathways, with the hypothalamus, neurotransmitters, and neuropeptides playing pivotal roles.^16,25–27^ The peripheral nervous system regulates skeletal metabolism via sensory neurons and autonomic neural networks. Sensory neurons, densely distributed in the periosteum, transmit signals to the CNS and have fewer branches within mineralized bone and BM. They affect osteogenic cells by secreting neurotransmitters and neuropeptides.^28^ The autonomic nervous system includes the SNS and the parasympathetic nervous system (PNS). Both branches participate in regulating bone remodeling, which affects osteogenesis and osteoclastogenesis.^29,30^ The SNS releases NE, which activates β-adrenergic receptors (β-ARs), inhibiting osteoblast differentiation and promoting osteoclast formation. Conversely, the PNS exerts antagonistic effects through cholinergic signaling, maintaining the homeostasis of bone remodeling.^30,31^

### Bidirectional regulatory pathways of the SNS in bone metabolism

The BBA is a dynamic bidirectional communication network, wherein SNS signaling serves as a critical pathway coordinating neuro-skeletal interactions.^32^ The SNS modulates bone metabolism through a neural fiber network predominantly composed of adrenergic fibers that release NE, supplemented by a smaller proportion of cholinergic fibers that release ACh.^33^ NE primarily inhibits osteogenesis and promotes osteoclastogenesis, constituting a key factor in the pathogenesis of osteoporosis.^11^ In contrast, cholinergic signaling exerts indirect bone-protective effects by regulating the BM microenvironment.^34^ Beyond classical neurotransmitters, dopamine (DA) plays pathological roles in OA, wherein dysregulated dopaminergic signaling facilitates the release of inflammatory cytokines and accelerates cartilage degradation.^35^ Neuropeptide Y (NPY) inhibits osteogenesis, influencing chondrocyte hypertrophy and extracellular matrix degradation, thereby promoting OA progression.^36^ As a vital mediator of the neuro-skeletal axis, leptin activates the hypothalamic-sympathetic pathway through central mechanisms to release NE, thereby suppressing bone formation. Leptin also directly modulates bone metabolic homeostasis through peripheral pathways.^37^ The synergistic and antagonistic interactions among these neurotransmitters form a complex regulatory network that maintains skeletal homeostasis within the BBA framework.

Similar to SNS efferent signaling, the skeletal system affects CNS function by secreting osteogenic factors, which can cross the BBB or act through indirect pathways, thereby forming an ascending feedback loop.^20^ Within brain structures, OCN interacts with various receptor subtypes distributed across different regions. BBB permeability allows OCN to inhibit gamma-aminobutyric acid (GABA) neurotransmission and selectively modulate neuronal subpopulations in the hippocampus, brainstem, and midbrain nuclei, thereby enhancing catecholamine and serotonin synthesis.^38^ The cerebrospinal fluid levels of OCN are decreased in a rat model of Parkinson’s disease (PD); however, the intracerebral administration of osteogenic OCN significantly promotes dopaminergic neuron survival via the AKT/GSK3β signaling pathway.^39^ LCN-2 exhibits pro-inflammatory effects in Alzheimer’s disease (AD) and PD, with LCN-2 knockout models demonstrating reduced neuroinflammation and brain injury.^40^ OPN deficiency impairs macrophage chemotaxis and the production of pro-inflammatory cytokines, whereas OPN presence stimulates myelination and remyelination processes, which are critical for repairing aberrant neuronal circuits in AD.^41,42^

**Fig. 1:**
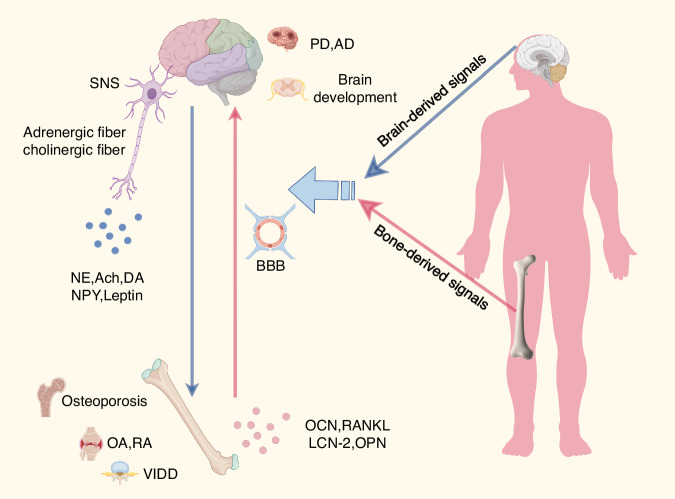
Bidirectional regulatory pathways of the SNS in bone metabolism. The diagram depicts the dynamic crosstalk between the central nervous system and skeletal tissue via sympathetic nervous signaling (SNS) and bone-derived factors. Left (brain-to-bone signaling): Brain-derived signals are transmitted primarily through adrenergic and cholinergic fibers, releasing neurotransmitters, including norepinephrine (NE), acetylcholine (ACh), dopamine (DA), neuropeptide Y (NPY), and leptin. These mediators regulate bone remodeling and contribute to the pathogenesis of osteoporosis, osteoarthritis (OA), rheumatoid arthritis (RA), and ventilation-induced diaphragm dysfunction (VIDD). Right (bone-to-brain signaling): Bone-derived factors, including osteocalcin (OCN), RANKL, lipocalin-2 (LCN-2), osteopontin (OPN), and other metabolites, cross the blood-brain barrier (BBB) to influence brain development and neural function. These signals are implicated in neurological disorders, including Parkinson’s disease (PD) and Alzheimer’s disease (AD)

In summary, the BBA, particularly its core component, the SNS, exerts a profound influence on bone metabolism by regulating neurofibers and various neurotransmitters (such as NE, DA, and NPY) and neuroendocrine factors (leptin). Overactivation and dysregulation of the SNS have been established as key pathogenic mechanisms contributing to osteoporosis, an increased risk of pathological fractures, and the development and progression of degenerative joint diseases such as OA and IVDD (Fig. 1).

## Anatomical features and molecular regulatory mechanisms of sympathetic innervation in osseous tissue

### Anatomical connection between the SNS and bone tissue

The skeletal system is not an isolated mechanical structure; its functions and homeostasis are intricately regulated by neural networks, including the SNS. Sympathetic nerve fibers exhibit specific spatial distribution patterns and temporal developmental trajectories within bone tissue. They are predominantly localized in two critical microenvironments: the periosteum, covering the bone surface, and the BM located within the diaphysis.^43^ These nerve fibers are also present within the nutrient canals traversing the mineralized bone matrix. The development of the autonomic innervation of long bones begins postnatally. In murine models, sympathetic fibers expressing NPY are first observed within the marrow cavity of the femur and tibia at postnatal day 6. By postnatal day 10, fibers co-expressing NPY and tyrosine hydroxylase (TH), the rate-limiting enzyme in catecholamine synthesis, become prominent in the epiphyseal regions and periarticular cartilage membranes.^33^ Regionally, TH^+^ SNS fibers demonstrate selective distribution, densely populating synovial membranes, subchondral bone, and the periosteum, but are generally absent from normal articular cartilage.^44^

Sympathetic nerve fibers innervating bone tissue can be classified into adrenergic fibers and cholinergic fibers based on the primary neurotransmitters released.^33^ However, the origin of cholinergic innervation to the skeletal system has been a subject of ongoing debate; whether it derives from the sympathetic or parasympathetic nervous system is controversial. An early investigation using retrograde neuronal tracing with viral vectors indicated that cholinergic signaling within the bone originates from parasympathetic innervation.^45^ However, recent evidence suggests that sacral autonomic outflow is sympathetic in nature.^46^ Furthermore, developmental studies have demonstrated that the ablation of sympathetic fibers in neonatal mice decreases both adrenergic and cholinergic fibers within the bone, supporting the hypothesis that skeletal cholinergic innervation is of sympathetic origin.^30^

In bone tissue, the sympathetic adrenergic nervous system primarily exerts its effects by releasing NE. NE binds to β2-ARs expressed on osteoblasts, thereby stimulating bone resorption and inhibiting osteogenesis.^11^ In contrast, cholinergic nerves release ACh. Cholinergic signaling is transmitted via osteogenic progenitor cells within the periosteum to the BM. This process regulates hematopoietic stem cell (HSC) and leukocyte migration^47^ and maintains HSC quiescence during hematopoietic regeneration.^48^ These findings suggest that the osteocytic network plays a critical role in mediating sympathetic noradrenergic signals to the BM to provide regulatory functions.^49^ Skeletal sympathetic cholinergic fibers exert anabolic effects on bone formation that are complementary to the central cholinergic suppression of sympathetic tone.^50^

### Molecular and cellular regulatory mechanisms

#### Norepinephrine

The stimulation of sympathetic nerve fibers induces the release of NE, which subsequently activates ARs on postsynaptic cells. These receptors are classified into two major G protein-coupled receptor (GPCR) subtypes: α-ARs and β-ARs. Notably, β-ARs predominate in skeletal tissue and mediate the sympathetic regulation of bone metabolism.^51^ Osteoblasts express three β-AR subtypes (β1, β2, and β3), which are also present on osteoclasts and MSCs.^52^ During MSC differentiation into osteoblasts, β-AR expression exhibits dynamic modulation.^53^ Mechanistically, β-AR activation inhibits osteogenic differentiation by engaging the canonical cAMP/PKA/CREB signaling pathway to suppress key transcription factors, whereas β-AR antagonists promote osteogenesis in MSCs. Specifically, β2-AR signaling modulates osteoclastogenesis through MSC-osteoblast communication, mediating ATF4 phosphorylation that upregulates RANKL expression, thereby facilitating osteoclast differentiation and bone resorption.^54,55^ Additionally, NE exerts dual regulatory effects on cartilage metabolism. β-AR-mediated signaling counteracts IL-1β-induced catabolic processes in chondrocytes and suppresses inflammatory responses.^56^

**Fig. 2:**
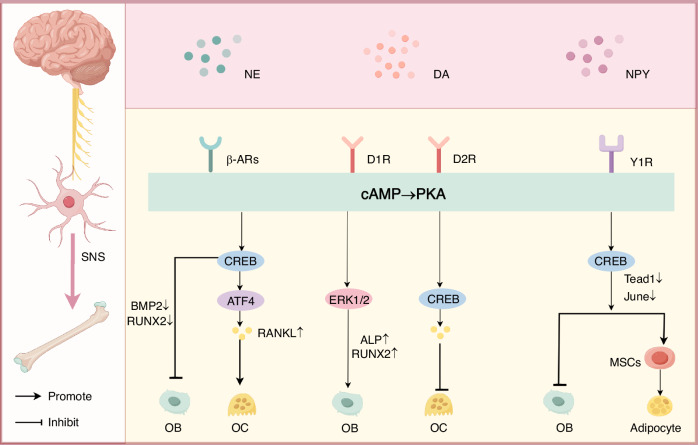
Molecular mechanisms of the sympathetic neurotransmitter-mediated regulation of osteoblast (OB) and osteoclast (OC) homeostasis. This schematic delineates the signaling pathways by which norepinephrine (NE), dopamine (DA), and neuropeptide Y (NPY) modulate bone remodeling through their respective receptors: β-adrenergic receptors (β-ARs), dopamine receptors D1R/D2R, and neuropeptide Y receptor Y1R. The key regulatory modules include the following: (1) NE/β-AR pathway: NE activates β-ARs, triggering the cAMP/PKA/CREB cascade, which suppresses osteoblast differentiation (by downregulating RUNX family transcription factor 2, RUNX2↓) and enhances osteoclast activity (by upregulating the receptor activator of nuclear factor kappa-B ligand, RANKL↑). (2) DA/D1R-D2R pathway: DA exerts bidirectional effects on bone metabolism: D1R activation promotes osteogenesis, while D2R signaling inhibits osteoclastogenesis, demonstrating receptor subtype-specific regulation. (3) NPY/Y1R pathway: NPY binding to Y1R inhibits the osteogenic differentiation of mesenchymal stem cells (MSCs), diverting their fate toward the adipocyte lineage (adipogenesis↑), thereby disrupting bone formation. Symbols: → activation; ⊣ inhibition

Unlike β-ARs, the expression levels of α-ARs (including α1 and α2 subtypes) are markedly lower in bone tissue, and their functional roles remain controversial.^44,57^ Although α-ARs have been detected in osteoblasts and osteoclasts, current evidence indicates that β-ARs serve as the primary mediators of SNS signaling in bone remodeling processes^51,58^ (Fig. 2).

#### Dopamine

DA regulates a variety of physiological processes, including sleep, motivation, reward, attention, autonomic motor control, vision, hormonal regulation, and motor function.^59^ Its dysregulation in metabolism is closely associated with osteopenia and osteoporosis, which is observed in neurodegenerative and psychiatric disorders such as PD and schizophrenia.^60^ DA exerts its biological effects through two GPCR subfamilies: D1-like receptors (D1R and D5R) and D2-like receptors (D2R, D3R, and D4R) expressed on osteoblasts and osteoclasts.^61^ DA influences bone metabolism through these receptor-mediated pathways.^62^ Specifically, the activation of D1R promotes osteogenic differentiation and mineralization via the cAMP/PKA signaling pathway, enhancing ERK1/2 phosphorylation and upregulating key osteogenic markers, such as RUNX2, alkaline phosphatase (ALP), and OPN.^63^ In contrast, D2R activation inhibits osteoclastogenesis through the cAMP/PKA/CREB pathway and involves RANK signaling.^64^ These findings suggest that DA modulates bone remodeling by regulating the functional activities of osteoblasts and osteoclasts, thereby playing a critical role in skeletal homeostasis.

DA can indirectly influence skeletal homeostasis through immunomodulatory mechanisms.^65^ It regulates immune cell activation, suppression, and proliferation via the D1R and D2R signaling pathways, thereby modulating their dynamic responses.^66,67^ For example, DA attenuates local joint inflammation by inhibiting NLRP3 inflammasome activation, leading to decreases in pro-inflammatory cytokines such as IL-1β and IL-18.^67^ DA can also suppress systemic inflammatory mediators, including TNF-α and IL-6, through central or peripheral pathways, indirectly protecting joint tissues from inflammatory damage.^65^ These immunoregulatory mechanisms offer novel insight into the multifaceted role of DA in bone metabolic disorders, highlighting its potential as a therapeutic target in osteoimmune regulation (Fig. 2).

#### Neuropeptide Y

NPY is a highly conserved 36-amino acid peptide predominantly enriched in the CNS, where it plays a critical role in regulating energy homeostasis, stress responses, and bone metabolism.^68^ The biological effects of NPY are mediated through five receptor subtypes (Y1R, Y2R, Y4R, Y5R, and Y6R), with Y1R and Y2R modulating skeletal metabolic processes.^69^

NPY modulates skeletal homeostasis through both central and peripheral pathways.^70^ Hypothalamic NPY signaling is crucial for the central regulation of bone formation.^71^ NPY centrally inhibits osteogenesis through hypothalamic Y2 receptors^72^ while peripherally activating osteoblastic Y1 receptors to coordinate bone formation with energy availability.^73^ The activation of NPY-Y1 receptors suppresses osteoblast differentiation and promotes the adipogenic commitment of MSCs, ultimately driving bone loss and pathological marrow adiposity.^68^ Mechanistically, NPY-Y1 signaling downregulates osteogenic transcription factors Tead1 and Jun through the cAMP/PKA/CREB pathway, shifting MSC fate toward adipogenesis. Conversely, NPY-Y2 receptor signaling centrally suppresses bone formation. Pharmacological Y2R antagonism enhances osteoblast differentiation and trabecular structuring, consequently elevating bone density through hypothalamic disinhibition^74^ (Fig. 2).

#### Leptin

Leptin is a hormone secreted by adipocytes that primarily exerts its effects by acting on the hypothalamic arcuate nucleus to suppress appetite and enhance energy expenditure.^75^ Leptin is also a pleiotropic cytokine and is involved in various physiological processes, including glucose metabolism, hematopoiesis, reproductive function, and immune system regulation.^76^

Leptin regulates bone metabolism through central and peripheral mechanisms. The central pathway involves leptin binding to its receptors in the hypothalamic arcuate nucleus, leading to activation of the SNS and the subsequent release of NE.^37^ NE released from sympathetic nerve fibers stimulates β-ARs on osteoblasts, inhibiting their proliferation and differentiation. Conversely, the peripheral mechanism involves direct interaction between leptin and LepR proteins expressed on osteoblasts and osteoclasts, modulating their activity and differentiation processes.^77,78^ Leptin also activates intracellular signaling cascades, such as JAK/STAT, PI3K/Akt, and MAPK pathways, influencing cell survival, mechanotransduction, and bone remodeling functions.^79^ Specifically, leptin promotes osteoblast proliferation and differentiation by activating the PI3K/Akt pathway while also regulating the OPG/RANKL ratio to suppress osteoclastogenesis and osteoclast activity.^80^ The peripheral actions of leptin support bone formation and inhibit resorption, which maintain skeletal homeostasis. However, the dysregulation of leptin levels, whether increased or decreased, can disrupt this balance, potentially contributing to the pathogenesis and progression of osteoporosis.

**Fig. 3:**
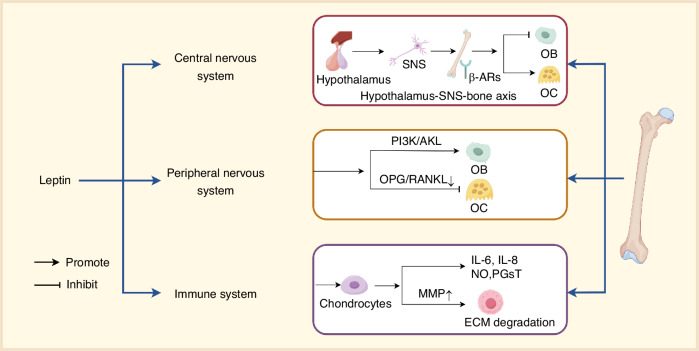
Central and peripheral mechanisms of the leptin-mediated regulation of bone metabolism and immune interactions. This figure summarizes the dual pathways through which leptin regulates bone homeostasis. Central pathway: Leptin binds to its receptor (LepR) in the hypothalamic arcuate nucleus, activating the sympathetic nervous system (SNS), which releases norepinephrine (NE). NE acts on osteoblasts (OB) through β-adrenergic receptors (β-ARs), suppressing OB differentiation. Peripheral pathway: Leptin promotes osteoblast proliferation and differentiation by activating the phosphatidylinositol 3-kinase/protein kinase B pathway (PI3K/Akt pathway), while also modulating the osteoprotegerin/receptor activator of nuclear factor kappa-B ligand ratio (OPG/RANKL ratio) to suppress osteoclastogenesis. Immune interplay: Leptin stimulates pro-inflammatory cytokines (IL-6 and IL-8), enhances macrophage/NK cell functions, and induces matrix metalloproteinases (MMPs) in chondrocytes, exacerbating extracellular matrix (ECM) degradation. Dysregulated leptin levels disrupt bone-immune system crosstalk, contributing to osteoporosis and inflammatory joint disorders. Symbols: → activation; ⊣ inhibition

Leptin also participates in innate and adaptive immune responses by stimulating the synthesis and secretion of pro-inflammatory cytokines, including IL-6, TNFα, and IL-12.^65^ It enhances macrophage phagocytic activity and promotes the proliferation of NK cells.^81^ Leptin also modulates the inflammatory microenvironment in joints, balances the dysregulation of catabolic and anabolic metabolic factors, and regulates skeletal and cartilage remodeling processes. In chondrocytes, leptin induces the production of pro-inflammatory mediators, such as IL-6, IL-8, nitric oxide (NO), and prostaglandins (PGs), while also upregulating the expression of matrix metalloproteinases (MMPs), leading to ECM degradation^82^ (Fig. 3).

## Pathophysiological mechanisms of the SNS in skeletal disorders

### Osteoporosis

**Fig. 4:**
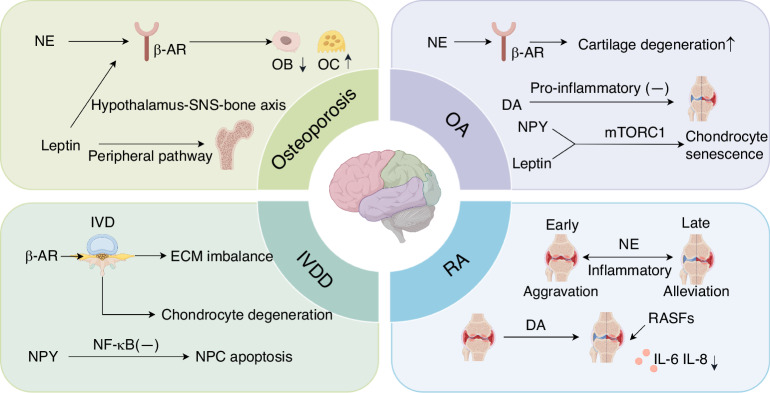
Sympathetic neurotransmitter-mediated regulatory mechanisms in bone-related diseases. This schematic delineates the role of sympathetic nervous system (SNS) signaling in osteoporosis, osteoarthritis (OA), rheumatoid arthritis (RA), and intervertebral disc degeneration (IVDD). Osteoporosis: Norepinephrine (NE) activates β-adrenergic receptors (β-ARs), suppressing osteoblast (OB) differentiation and promoting osteoclast (OC) activity. Leptin exacerbates bone loss via the hypothalamus-SNS-bone axis, decreasing the osteoprotegerin (OPG)/RANKL ratio. OA: β2-AR signaling inhibits chondrocyte proliferation and induces apoptosis, accelerating cartilage degeneration. Dopamine (DA) alleviates inflammation through anti-inflammatory pathways. Neuropeptide Y (NPY) and leptin activate the mechanistic target of rapamycin complex 1 (mTORC1), driving chondrocyte senescence. RA: Early phase: The SNS hyperactivity aggravates synovitis via NE-mediated pathways. Late phase: NE signaling reduces inflammation. DA suppresses IL-6 and IL-8 secretion in rheumatoid arthritis synovial fibroblasts (RASFs), mitigating inflammation. IVDD: β-AR activation disrupts extracellular matrix (ECM) homeostasis, promoting disc degeneration. NPY inhibits the NF-κB pathway, attenuating nucleus pulposus cell (NPC) apoptosis. Symbols: ↑ activation or increase; ↓ inhibition or decrease; and (−) suppression

Osteoporosis is a prevalent skeletal disorder characterized by decreased bone mineral density and increased susceptibility to fractures.^83^ This systemic condition manifests as a reduction in bone mass, the deterioration of microarchitectural integrity, and increased bone fragility.^84^ The disease predominantly affects the elderly population^85^ (Fig. 4).

The central signals generated by the hypothalamus are transmitted to the sympathetic nerve fibers of the bone tissue through the spinal cord, which promotes the coordinated release of NE and NPY in the bone microenvironment and forms the initial signal of bone metabolic imbalance. NE inhibits osteoblast-mediated bone formation by activating β2-AR on osteocytes while promoting osteoclast-driven bone resorption. These effects break the dynamic balance between bone formation and bone resorption.^33,86^ Wu et al. confirmed that inhibiting sympathetic nerve activity could promote the osteogenic differentiation ability of osteoblasts and MSCs.^87^ Wang et al. reported that the long-term use of adrenaline drugs increases the chance of fracture. They also found that the β2-AR agonist terbutaline directly inhibits bone formation by impairing osteogenic differentiation and mineralization.^88^ Sui et al. reported that β2AR agonists release RANKL through osteoblasts, activate the downstream signal of osteoclast β2AR, and significantly promote bone resorption by upregulating osteoclast miR-21.^89^ Studies have shown that β-AR upregulation drives bone resorption by enhancing RANKL signaling, but the downregulation of α-AR leads to the loss of sympathetic nerve inhibition function, which together aggravates heart failure-related bone loss.^86^

Notably, the SNS regulation of osteoporosis is not unidirectional. Although adrenergic nerve fibers predominantly drive bone loss, studies have demonstrated that cholinergic nerve fibers derived from the SNS and their secreted neuromodulatory protein, neuromodulin (NRTN), play an active anabolic role in bone remodeling. In vitro, NRTN induces the differentiation of bone marrow stem cells (BMSCs) into osteoblasts. NRTN was shown to improve bone microstructure and increase bone mass in an ovariectomized (OVX) mouse model of osteoporosis, indicating an intrinsic balancing mechanism within the SNS that regulates bone homeostasis.^34^

NPY serves as a modulator of bone homeostasis and is capable of inhibiting the proliferation and differentiation of osteoblasts while promoting osteoclastogenesis.^90^ Experimental evidence supports this pathological role. The specific knockout of NPY derived from MSCs significantly enhanced cortical bone strength in rats and upregulated the expression of osteogenic markers, such as bone sialoprotein and osteocalcin in BMSCs, effectively driving their osteogenic differentiation.^91^ Other research demonstrated that NPY1 receptor antagonists facilitated the repair of osteoporotic microdamage and enhanced the osteogenic differentiation of BMSCs.^92^ Melatonin promoted fracture healing in a rat femoral fracture model and increased the local expression of NPY/NPY1R, suggesting that melatonin may enhance the osteogenic differentiation of MSCs and fracture repair by activating the NPY/NPY1R signaling pathway. This reveals the potential pro-osteogenic role of this pathway under specific therapeutic interventions.^93^ NPY2R expression was significantly increased in an OVX-induced osteoporosis model, and its antagonist, which is capable of crossing the BBB, targeted the hypothalamic Y2 receptor to inhibit NPY release, thereby increasing bone density and mitigating bone loss.^74^ Collectively, these findings underscore the significance of NPY signaling dysregulation in the pathogenesis of osteoporosis.

The role of leptin in osteoporosis depends on the balance between its direct and indirect skeletal effects.^80^ Leptin inhibits bone formation through centrally mediated pathways while directly promoting osteogenesis through peripheral mechanisms.^94–96^ Alterations in leptin signaling exert differential effects on axial bones, appendicular bones, cortical bone, and trabecular bone, with leptin primarily associated with bone remodeling in OVX rats exhibiting bone loss.^78^ Within this central regulatory pathway, NPY acts as a key downstream effector. In obesity, leptin resistance alleviates the normal suppression of NPY, triggering its excessive release and consequent potent inhibition of bone formation. Studies have confirmed that the denervation of retroperitoneal white adipose tissue selectively reduces NPY levels (without altering leptin itself), thereby reversing this process.^71^ During aging, decreasing sex hormone levels and increasing serum leptin are associated with reduced leptin receptor expression, decreases in osteogenic markers, and increases in osteoclastic markers, resulting in a net increase in bone resorption versus formation. Research has demonstrated that Qing’e Decoction corrects age-related bone loss and modulates bone metabolic equilibrium by enhancing sex hormone levels and leptin receptor expression.^97^ Notably, both hyperleptinemia and leptin deficiency are implicated in the pathogenesis of osteoporosis, underscoring the critical importance of maintaining physiological leptin homeostasis^80^ (Table 1).

**Table 1:** Mechanisms of action mediated by the SNS in osteoporosis

Experimental model	Bone cell lineage	Key Factors	Intervention	Actions and their mechanisms	Ref.
In vivo: SD rats	OB, OC	NE	Chemical sympathectomy; guanethidine (40 mg/kg/day, i.p.) for 5 weeks	NE promotes osteoclastogenesis by activating β-AR, upregulating RANKL expression. NE also suppresses osteogenesis by inhibiting α-AR, decreasing OPG and osteocalcin expression.	^86^
In vivo: OVX mice; in vitro: BMSCs	BMSCs, OB, OC	ACh	In vivo: NRTN injections (0.5, 1, 2 mg/kg, i.v.) twice weekly for 4 weeks; in vitro: NRTN added to osteogenic medium	NRTN directly promotes the osteoblastic differentiation of BMSCs without affecting their proliferation or osteoclast differentiation.	^34^
In vivo: SD rats; in vitro: BMSCs	BMSCs, OB, OC	NPY	In vivo: MLT; NPY1R inhibitor (20 μg/rat/q2d); in vitro: MLT (2 mmol/L) ± NPY1R inhibitor (0.1 mmol/L) in MSC medium	MLT activates NPY/NPY1R signaling, promoting MSC proliferation, migration, and osteogenic differentiation.	^93^
In vivo: OVX mice; in vitro: Bone marrow cells	OB, OC	NPY	In vivo: Y2 receptor antagonist i.p.; in vitro: Y2 receptor antagonist in culture medium	Y2 receptor antagonist suppresses hypothalamic Y2 receptors, leading to the centrally mediated promotion of osteogenesis and the inhibition of osteoclastogenesis.	^74^
In vivo: *Adrb2*^*−*^^*/*^^*−*^ (β_2_-AR KO), *Dbh*^*−*^^*/*^^*−*^ (DBH KO), ob/ob (leptin-def.), *Cart*^*−*^^*/*^^*−*^ (CART KO) mice; in vitro: OB, OC, BMCs	OB, OC	Leptin	In vivo: ICV; OVX; in vitro: ISO; PKA inhibitor; ATF4 mutant transfection	Sympathetic activation via β_2_-AR inhibits osteoclast differentiation. Leptin suppresses osteogenesis via the hypothalamus-sympathetic nerve axis. CART peptide antagonizes bone resorption.	^94^
In vivo: naturally aged SD rats	OB, OC	Leptin	Qing’e Formula extract	Extract enhances leptin signaling, ameliorates the imbalance between osteogenesis and bone resorption, and increases BMD.	^97^

*ACh* acetylcholine, *BMSCs* bone marrow mesenchymal stem cells, *BMC* bone marrow cells, *CART* cocaine- and amphetamine-regulated transcript, *ICV* intracerebroventricular, *ISO* isoproterenol, *MLT* melatonin, *NE* norepinephrine, *NRTN* neurturin, *NPY* neuropeptide Y, *NPY1R* neuropeptide Y receptor type 1, *OB* Osteoblast, *OC* osteoclast, *OVX* ovariectomized, *PKA* protein kinase A, *RANKL* receptor activator of nuclear factor kappa-B ligand, *Y2R* neuropeptide Y receptor type 2

### Osteoarthritis

OA, a degenerative joint disorder characterized by progressive cartilage deterioration, primarily affects weight-bearing joints, including the knees, hips, and spine. The clinical manifestations include pain, stiffness, and restricted mobility, with pathogenesis stemming from mechanical friction between exposed subchondral bones following articular cartilage destruction.^98,99^ Traditional medicine classifies this condition as a “wear-and-tear disease” initiated by cartilage degradation.^100^ The OA pathological cascade involves inflammatory responses triggered by complex signaling crosstalk between chondrocytes and osteoblasts, as well as the dysregulation of inflammatory mediators and aberrant signal transduction pathways within the joint microenvironment^101,102^ (Fig. 4).

The neuromodulatory theory of OA, initially proposed over 130 years ago, has received substantial empirical validation.^103,104^ Recent studies have demonstrated that peripheral sympathetic nerve fibers participate in ECM deposition during endochondral ossification.^105^ The immunodetection of AR subtypes and TH in chondrocytes reveals that low NE concentrations reverse IL-1β-induced matrix degradation and inflammation and decrease cell proliferation via β-AR signaling. Conversely, high NE concentrations increase cell proliferation and induce apoptosis via α1-AR signaling.^106^ NE binding to ARs inhibits chondrocyte proliferation while promoting apoptosis, potentially accelerating cartilage degeneration.^56^ Karima et al. reported that NE significantly suppressed aggrecan and type II collagen expression in adipose-derived stem cells, inhibiting chondrogenesis. This effect was abolished by the β2-AR antagonist yohimbine.^107^ Zsuza et al. further demonstrated NE’s dose-dependent inhibition of chondrogenesis and promotion of hypertrophic differentiation.^108^ Lu et al. established the chondroprotective and anti-inflammatory effects of DA on IL-1β-stimulated chondrocytes.^35^

Notably, the articular cartilage of normal adults lacks innervation. However, significant pathological nerve infiltration occurs during OA progression. Suri and colleagues reported the localization of sensory nerve fibers (SP^+^, CGRP^+^) and sympathetic nerve fibers (NPY^+^) in the articular cartilage of human tibial femoral arthritis.^109^ During OA development, NPY^+^ sympathetic fibers may grow into the cartilage through the blood vessels of the subchondral BM and release NPY into synovial fluid to affect cartilage metabolism.^110^ NPY concentrations in synovial fluid are increased in middle and late OA patients compared with early OA patients.^111,112^ In the OA model, the intraarticular administration of NPY can manifest as increased cartilage matrix degradation, increased chondrocyte apoptosis, and enhanced inflammatory response.^36^ Kang et al. showed that NPY triggers the mTORC1 signaling pathway by activating NPY2R on articular chondrocytes, which stimulates chondrocyte hypertrophy and cartilage matrix degradation and accelerates OA progression.^113^ Together, these findings establish the key role of NPY signaling pathway dysregulation in OA cartilage catabolism.

Leptin stimulates inflammatory cytokine production in OA synovial fibroblasts. Pearson et al. demonstrated that synovial fibroblasts from patients with obesity and OA secrete elevated levels of pro-inflammatory IL-6. While leptin alone fails to directly stimulate IL-6 secretion in isolated fibroblasts, it potentiates chondrocyte-fibroblast interactions, thereby synergistically enhancing pro-inflammatory mediator release.^114^ Leptin activates the IRS-1/PI3K/Akt/AP-1 signaling pathway in synovial fibroblasts by binding to the Ob-Rb receptor, markedly upregulating IL-6 and IL-8 synthesis.^115,116^ Leptin and NPY exhibit distinct yet overlapping roles in OA progression. Studies have indicated that mTOR knockdown in mice increases autophagy marker expression, reduces chondrocyte apoptosis, and suppresses catabolic factor MMP-13 expression.^117^ Zhao et al. revealed that physiological leptin doses induce chondrocyte senescence following Ob-Rb overexpression. High leptin concentrations inhibit autophagy by activating the mTOR pathway. The blockade of mTOR signaling rescues leptin-induced autophagic flux in chondrocytes and partially attenuates senescence.^118^

### Rheumatoid arthritis

RA is a chronic systemic autoimmune disorder characterized by inflammatory synovitis. It predominantly affects peripheral joints and the associated periarticular soft tissues.^119^ Pathological alterations in synovial tissue, including ectopic lymphoid neogenesis, pannus formation, aberrant synovial hyperplasia, and extensive inflammatory cell infiltration, are pivotal drivers of RA progression.^120,121^ Within this microenvironment, activated fibroblast-like synoviocytes (RA-FLS) emerge as pivotal cellular mediators of synovial transformation from homeostasis to destructive pannus, demonstrating quasi-malignant invasive properties and pathological cytokine hypersecretion^122,123^ (Fig. 4).

The SNS and its neurotransmitters play a dual role in the pathogenesis and progression of RA. In murine models of experimental arthritis, prior sympathetic neurectomy at an early stage ameliorates disease severity, whereas neurectomy performed at a later phase exacerbates RA symptoms.^124^ This discrepancy may be attributed to the elimination of TH^+^ cells, as well as anti-inflammatory properties that emerge during the course of RA.^125^ Evidence indicates that catecholaminergic cells expressing TH contribute to the anti-inflammatory responses in arthritis treatment. Sympathetic nerve fibers synthesize and release catecholamines through the key enzyme TH and mediate signaling through neurotransmitter release at peripheral nerve terminals.^104^ Neurotransmitters, such as NE, generally exert anti-inflammatory effects.^44^ At elevated concentrations, NE can suppress the production of inflammatory cytokines, including TNF and IL-12, by activating β2-AR, thereby attenuating inflammation.^126^

DA has been shown to play a regulatory role in the pathological processes of RA. Nie et al. reported that local DA synthesis occurs in RA synovial cells, particularly in fibroblasts, macrophages, and B cells, and suggested that the synovial dopaminergic system may contribute to fibroblast migration in arthritic joints, thereby attenuating the progression of joint damage in RA patients.^127,128^ Capellino et al. demonstrated that the DA-mediated activation of DR reduces the secretion of IL-6 and IL-8 by synovial fibroblasts in treatment-naïve patients with RA, an effect that remains unaffected by anti-rheumatic drug interventions.^129^ Additional studies have indicated that inhibiting either D1-like or D2-like DR activation ameliorates collagen-induced arthritis in mouse models.^130,131^ Beyond synovial inflammation, DA is also implicated in systemic osteoporosis associated with RA. DA, synthesized and used locally in bone tissue via paracrine/autocrine mechanisms, exerts a dual protective effect against RA-related bone loss: it promotes osteoblast-mediated bone mineralization by activating D2-like receptors while simultaneously inhibiting osteoclast-mediated bone resorption^132^ (Table 2).

**Table 2:** Regulatory roles of the SNS in arthritis

Experimental model	Bone cell lineage	Key factors	Intervention	Actions and their mechanisms	Ref.
In vitro: chondrocytes	ACs	NE	NE treatment (10^−6^ mol/L and 10^−8^ mol/L); IL-1β (0.5 ng/mL) to simulate inflammation	High NE concentrations inhibit IL-1β-induced IL-8 and MMP-13 expression, reverse GAG and type II collagen reduction, and reduce proliferation. Low NE concentrations induce apoptosis and promote proliferation.	^106^
In vitro: chondrocytes; in vivo: mouse OA	ACs	DA	In vitro: DA (25, 50, 100 μmol/L); IL-1β In vivo: IA injection of DA weekly for 12 weeks post-OA induction	DA inhibits IL-1β-induced NF-κB signaling and JAK2/STAT3 phosphorylation, exerting anti-inflammatory and anti-catabolic effects, protecting chondrocytes.	^35^
In vitro: chondrocytes	ACs	NE	NE and β-AR antagonist (0.1, 1.0, 10 μg/mL); IL-1β	β-AR signaling reduces MMPs expression and increases matrix protein synthesis by inhibiting JNK/ERK/NF-κB pathways.	^56^
In vivo: SD rats	ACs	NPY	No external intervention; observation at P1/P5/P10 time points during natural cartilage development	Nerve fibers gradually degrade during cartilage development, accompanied by increased SP and NPY release.	^110^
In vitro: chondrocytes; in vivo: mouse OA	ACs	NPY	In vitro: NPY (0–100 nmol/L); NPY2R antagonist (1 μmol/L); mTORC1 inhibitor rapamycin (50 nmol/L) In vivo: IA injection of NPY (10 μmol/L, 10 μL/injection, 1x/week × 8 weeks) or NPY2R antagonist (1 μmol/L)	NPY promotes chondrocyte hypertrophy and cartilage degradation via NPY2R-mediated activation of the mTORC1 signaling pathway.	^36^
In vitro: SFs	SFs	Leptin	Leptin (0.1–3 μmol/L); signaling pathway inhibitors	Leptin induces IL-6 expression in human SFs via the OBRl receptor signaling pathway.	^115^
In vitro: SFs	SFs	Leptin	Leptin (0.1–3 μmol/L); signaling pathway inhibitors	Leptin induces IL-8 expression in human SFs via the leptin receptor, IRS-1, PI3K, and Akt cascade and promotes NF-κB/p300 binding.	^116^
In vitro: chondrocytes	ACs	Leptin	Leptin (0, 10, 100, 200 ng/mL); mTOR inhibitor rapamycin; Ob-Rb overexpression	High Ob-Rb expression enhances leptin signaling sensitivity, activating the mTOR pathway, inhibiting autophagy, and inducing cell senescence.	^118^
In vivo: CIA mice, adjuvant rats; in vitro: immune cells	None	NE, NPY	In vivo: sympathectomy; NE treatment (10^−6^ mol/L and 10^−8^ mol/L)	SNS plays a pro-inflammatory role early by promoting Th1/Th17 responses, antibody production, and a late anti-inflammatory response through the appearance of Tr1^+^ cells and β2-AR-mediated inflammation inhibition.	^104^
In vitro: SFs	SFs	DA	DA treatment (10^−9^ to 10^−5^ mol/L)	DA inhibits IL-6 and IL-8 secretion via D_1_-like receptors.	^129^
In vivo: CIA mice In vitro: macrophages	OC	DA	In vivo: D1R antagonist: (0.005 mg/kg or 0.05 mg/kg), every other day; D1R agonist: i (0.2–2.0 mg/kg)	D1R antagonist ameliorates arthritis severity and inhibits osteoclast differentiation by suppressing D1R.	^130^
In vivo: CIA mice, *Drd2*^*−*^^*/*^^*−*^, *Drd1*^*−*^^*/*^^*−*^ mice	None	DA	In vivo: D2R agonist (quinpirole, 0.3 mg/kg, IP, 2x/week × 3 weeks)	D2R agonist alleviates arthritis by downregulating Th17 cytokines and upregulating Treg cytokines.	^131^
In vitro: OB, OC	OB, OC	DA	D1-like receptor agonist (10^−6^ to 10^−8^ mol/L); D2-like receptor agonist (10^−6^ to 10^−8^ mol/L)	DA promotes bone formation via D2-like receptors in RA without affecting bone resorption.	^132^

*ACs* articular chondrocytes, *AR* adrenergic receptor, *CIA* collagen-induced arthritis, *DA* dopamine, *Drd1*^−^^/^^−^ dopamine receptor D1 knockout, *Drd2*^−^^/−^ dopamine receptor D2 knockout, *IA* intra-articular, *IL* Interleukin, *JAK/STAT* janus kinase/signal transducer and activator of transcription, *MMP* matrix metalloproteinase, *mTOR* mammalian target of rapamycin, *NF-κB* nuclear factor kappa B, *NPY* Neuropeptide Y, *NPY2R* neuropeptide Y receptor type 2, *OA* Osteoarthritis, *OB-Rb* long form of leptin receptor, *Rapamycin* mTOR inhibitor, *SFs* synovial fibroblasts, *Th* T helper cell, *Treg* regulatory T cell

### Intervertebral disc degeneration

IVDD is a common and multifactorial condition characterized by the progressive deterioration of the disc structure and biomechanical function, primarily resulting from the loss of hydration, which leads to reduced disc height.^98^ The disc consists of three distinct anatomical regions: the gelatinous nucleus pulposus (NP), the concentric lamellae of the annulus fibrosus (AF), and the cartilaginous endplate (EP). The EP anchors the NP-AF complex to the vertebral bodies.^133^ In a healthy state, the NP is rich in proteoglycan-based extracellular matrix (ECM), which is predominantly composed of type II collagen and aggrecan in a homeostatic state maintained by NP cells.^134^ Pathologically, IVDD involves dysregulated ECM metabolism and cellular dysfunction within both the AF and NP compartments, ultimately leading to chronic low back pain, particularly among the elderly population^135^ (Fig. 4).

Anatomically, the sinuvertebral nerve (SVN), which provides the principal innervation to the intervertebral disc, is composed of somatic sensory branches from the lateral division of the spinal nerve and postganglionic sympathetic fibers originating from the gray rami communicantes.^136^ This composite structure supplies the disc with dual proprioception and nociception innervations, facilitating the transmission of mechanical sensation and pain signals.^137^ Studies have indicated that the SVN serves as a critical neuroanatomical interface between sympathetic efferent pathways and spinal cord neural circuits.^138^ Clinical data further reveal a significant correlation between increased noradrenergic sympathetic fiber density and enhanced pain signaling in patients with IVDD, whereas the modulation of neuropeptide levels has been shown to alleviate pain associated with disc degeneration.^139^

Studies have revealed that sympathetic nerve fibers and ARs are present in both healthy and degenerated intervertebral disc tissues. The progression of disc degeneration is further associated with increased subchondral bone sclerosis and EP damage. The significant upregulation of α1-AR, α2-AR, and β2-AR expression has been observed in patients with vertebral EP inflammation and structural alterations.^140^ Articular cartilage in OA and disc cartilage share similarities as weight-bearing tissues and exhibit parallel changes during degeneration.^141^ Research has shown that β2-AR expression is enhanced and more widely distributed in severely degenerated intervertebral discs in mice. In degenerated disc tissues, the abnormal activation of β2-ARs is significantly positively correlated with the pathological grade of IVDD as well as an imbalance in extracellular matrix metabolism, characterized by the degradation of type II collagen and the loss of proteoglycans.^142^

Compared with naturally aged intervertebral discs, degenerated discs exhibit significantly upregulated gene expression levels of NPY and its receptors Y1R/Y2R, indicating that neuropeptide signaling plays a crucial role in the disc degeneration process.^143^ Further investigations have revealed that under pathological stimuli such as inflammatory factors (e.g., IL-1β) and abnormal mechanical stress, NPY synthesis is increased and Y1R expression is upregulated in AF cells.^144^ NPY demonstrates protective effects against degeneration. A study by Sun et al. showed that in an in vitro IL-1β-induced model of IDD, NPY attenuates nucleus pulposus cell apoptosis by suppressing the mitochondrial apoptotic pathway and downregulates the expression of catabolic proteases (e.g., MMPs) involved in ECM breakdown, thereby facilitating delayed ECM destruction^145,146^ (Table 3).

**Table 3:** Role of the SNS in intervertebral disc degeneration

Experimental model	Bone cell lineage	Key factors	Intervention	Actions and their mechanisms	Ref.
In vivo: large domestic pigs	None	NE NPY	Injection of retrograde tracer and contrast agent into L_4_–L_5_ IVD; laser ablation to induce NP dehydration	Pain signaling is modulated through the release of NE and various neuropeptides.	^139^
In vivo: human IVD tissue, WT (C57BL/6) and SM/J mice; in vitro: IVD cells	IVD cells	NE	In vitro: NE treatment (10^−8^ mol/L or 10^−6^ mol/L) for 5 or 15 min	β2-AR expression is upregulated in degenerative IVD and correlates with extracellular matrix ECM alterations.	^142^
In vitro: NP cells; in vivo: human IVD tissue	NP cells	NPY	IL-1β (30 ng/mL) to induce injury; NPY treatment (10^−12^ to 10^−8^ mol/L)	NPY primarily acts via the Y2 receptor; Y2R expression is upregulated in degenerated IVD and IL-1β-stimulated NP cells.	^143^
In vitro: AF cells	AF cells	NPY	Mechanical stress; inflammatory stimulation (IL-1β); combined: mechanical stress + IL-1β	NPY expression increases in AF cells under inflammatory and mechanical stress; NPY may participate in pain modulation via NPY-1R.	^144^
In vivo: human degenerated IVD tissue; in vitro: NP cells	NP cells	NPY	In vitro: NPY (10^−12^ to 10^−8^ mol/L) for 24, 48, 72 h; pretreatment with NPY (10^−10^ mol/L), then co-culture with IL-1β (30 ng/mL)	NPY exerts anti-apoptotic effects, promotes proliferation, and inhibits ECM degradation.	^145^

*AF* annulus fibrosus, *ECM* extracellular matrix, *IL-1β* Interleukin-1 Beta, *IVD* intervertebral disc, *NP* nucleus pulposus, *NE* norepinephrine, *NPY* neuropeptide Y, *NPY1R* neuropeptide Y receptor type 1, *Y2R* neuropeptide Y receptor type 2, *WT* wild type, *SM/J* small mouse/J strain

## Novel treatment for bone-related diseases based on the BBA

Innovative therapeutic strategies targeting the SNS demonstrate breakthrough efficacy in orthopedic disorders through the synergistic modulation of neural receptor signaling pathways. The co-administration of propranolol and parathyroid hormone (PTH) synergistically enhances systemic bone formation and accelerates osteoporotic fracture healing. In murine osteoporotic fracture models, propranolol augments PTH’s osteogenic effects by approximately 80%. Mechanistically, propranolol antagonizes the neuronal ACh-mediated suppression of PTH-induced osteoblast differentiation via β-AR blockade, thereby restoring the expression of osteogenic transcription factors Bmal1 and Runx2.^147^ Intra-articular delivery systems, including hydrogels, exosomes, liposomes, or polyethylene glycol microspheres encapsulating β2-AR antagonists, enable targeted application to osteoarthritic joints, promoting cartilage remodeling while slowing OA progression.^148,149^ Concurrently, DRs have emerged as novel regulatory targets. D1 receptor agonists enhance osteoblast differentiation via ERK1/2 phosphorylation pathways, counteracting glucocorticoid-induced bone loss,^63^ whereas D2-like DRs potentiate osteoblast mineralization capacity and selectively induce osteoclastogenesis in rheumatoid arthritis microenvironments without altering osteoclast activation or resorption processes.^132^ These findings indicate that the precise neuromodulation of receptor signaling pathways can reconfigure skeletal metabolic equilibrium, establishing targeted pharmacological approaches for osteoporosis and arthritis.

Cross-system integrated therapeutic strategies target the pathological interplay between sympathetic nerves, immunity, and the gut microbiota. Research has revealed that NPY overexpression in ovariectomized rat models induces gut dysbiosis, exacerbates colonic inflammation, and increases intestinal permeability, facilitating the systemic translocation of gut metabolites, such as lipopolysaccharide (LPS). Circulating LPS triggers osteoblast pyroptosis and suppresses differentiation, aggravating osteoporosis, which is a pathological state reversible by Y1R antagonism.^150^ Leptin, a pivotal regulator of the neuro-metabolic-osteoarticular axis, disrupts chondrocyte homeostasis in OA by activating the mTOR pathway, promoting cellular senescence and matrix degradation.^118^ As a therapeutic target, leptin not only potentially mitigates cartilage degeneration but also elucidates the molecular mechanism whereby weight loss alleviates OA symptoms in patients with obesity.^151^

The regulation of bone by sympathetic nerves using exogenous electrical stimulation techniques, along with the application of smart materials, represents a promising frontier in bone tissue engineering. In mid-stage arthritis, electroacupuncture selectively elevates local NE levels in the synovium without increasing systemic NE concentrations, which inhibits pro-inflammatory factor release, alleviates cartilage damage, and reduces pain.^152^ In beagle dog implant models, microelectrode stimulation of sympathetic nerves significantly enhances bone formation and osseointegration by dilating local vasculature and improving microcirculation.^153^ However, synchronized electrical and tissue-damage signals may overactivate sympathetic nerves, leading to excessive NE release that impedes the osteogenic differentiation of MSCs via β_2_-AR signaling. Researchers developed a composite carrier ZnS@PDA-PRO to address this paradox. In this system, the piezoelectric zinc sulfide (ZnS) core generates electrical signals under mechanical stress while releasing Zn^2+^ to promote osteogenic differentiation. The polydopamine (PDA) coating provides a biomimetic interface for enhanced cell adhesion, and the encapsulated β-AR blocker propranolol (PRO) mitigates sympathetic inhibition by blocking β_2_-AR signaling. These three components synergistically increase bone regeneration efficiency in critical defect models.^154^ Collectively, these mechanisms form a multidimensional “electrical signal–nerve–bone metabolism” regulatory network, providing a theoretical foundation for sympathetic nerve-targeted bone regeneration strategies.

The indirect modulation of sympathetic tone by endogenous ionic signals represents a highly promising emerging strategy. Studies have demonstrated that divalent cations (Mg^2+^, Zn^2+^, and Cu^2+^) can indirectly regulate sympathetic nerve tension by activating the skeletal interoception pathway. These ions stimulate macrophages to secrete prostaglandin E2 (PGE2), which binds to EP4 receptors on intrabony sensory neurons. This binding transmits inflammatory signals to central regions, such as the hypothalamic ventromedial nucleus, triggering a CREB phosphorylation cascade that downregulates sympathetic output. This process decreases the density of TH^+^ nerve fibers in the periosteum and circulating epinephrine levels. Ultimately, this pathway alleviates the sympathetic inhibition of osteogenic activity, creating a favorable neuro-microenvironment for bone regeneration.^155^

**Fig. 5:**
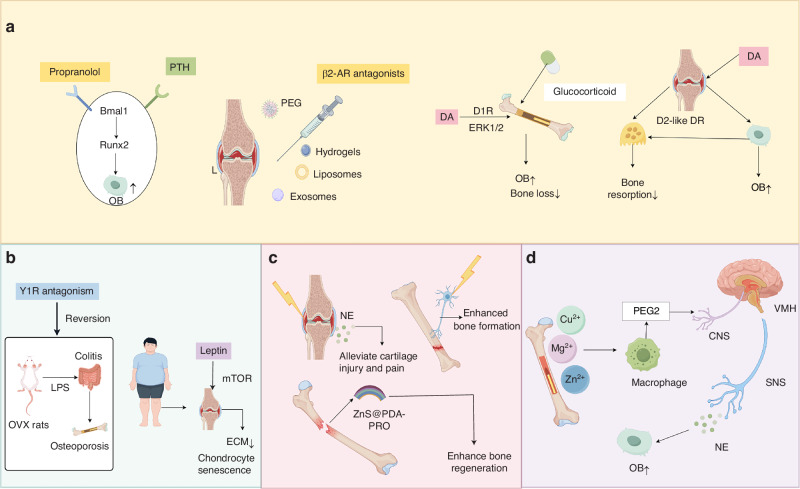
Novel therapeutic strategies targeting the brain-bone axis (BBA) for bone-related disorders. **a** Pharmacological modulation of neural receptor pathways: The systemic co-administration of propranolol (β_2_-AR antagonist) and parathyroid hormone (PTH) synergistically enhances osteogenesis by antagonizing the neuronal acetylcholine-mediated suppression of osteoblast differentiation, restoring the expression of Bmal1 and Runx2. Localized delivery systems (e.g., hydrogels, liposomes, exosomes, and PEG microspheres) enable the targeted application of β_2_-AR antagonists to osteoarthritic joints, promoting cartilage remodeling. Dopamine receptors (DRs) serve as regulatory targets: D1R activation via ERK1/2 phosphorylation counteracts glucocorticoid-induced bone loss, while D2-like DRs modulate osteoblast mineralization and osteoclastogenesis in rheumatoid arthritis. **b** Targeting the neuro-immune-microbial axis in bone metabolic diseases: NPY overexpression induces gut dysbiosis, increases intestinal permeability, and facilitates systemic LPS translocation, triggering osteoblast pyroptosis and impairing differentiation (OVX rat model illustrates osteoporotic pathology). Y1R blockade mitigates these effects and restores bone homeostasis. Leptin disrupts chondrocyte homeostasis by activating mTOR, which promotes senescence and matrix degradation in osteoarthritis. **c** Smart material-mediated electrostimulation for sympathetic neuromodulation:Electroacupuncture increases local synovial NE levels to suppress inflammation; microelectrode stimulation dilates local vasculature to enhance osseointegration. The ZnS@PDA-PRO composite integrates the following: (1) a piezoelectric ZnS core (generating electrical signals and releasing osteogenic Zn^2+^ under mechanical stress), (2) a polydopamine (PDA) coating for biomimetic cell adhesion, and (3) encapsulated propranolol (PRO) to block β_2_-AR signaling and mitigate sympathetic inhibition. These components synergistically enhance critical bone defect regeneration. **d** Endogenous ion-mediated sympathetic regulation: Divalent cations (Mg^2+^, Zn^2+^, and Cu^2+^) activate skeletal interoception pathways, stimulating macrophage-derived PGE_2_, which binds EP4 receptors on sensory neurons. This reduces hypothalamic sympathetic output through CREB phosphorylation in the ventromedial nucleus (VMH), which manifests as decreased periosteal TH^+^ nerve fiber density and circulating epinephrine levels, ultimately alleviating the sympathetic suppression of osteogenesis

Innovative therapeutic strategies based on the BBA framework have pioneered diverse pathways for managing orthopedic diseases. These approaches encompass precise interventions in neural receptor signaling pathways, the integration of cross-system pathological networks, the optimization of neuromodulation techniques, and indirect regulation through endogenous ion signaling. Such groundbreaking advances deepen our understanding of skeletal metabolism and the pathological mechanisms underlying bone-joint disorders and establish a robust foundation for developing more efficacious treatment regimens (Fig. 5).

## Limitations and future research directions

The current study systematically elucidates the molecular mechanisms by which the SNS regulates bone metabolism through neurotransmitters and its role in bone-related diseases. However, several limitations persist. First, current research primarily relies on animal models and in vitro experiments, lacking large-scale clinical data to support its translational potential. Second, while existing work focuses predominantly on the unidirectional regulatory role of the SNS, the synergistic or antagonistic interplay between the SNS and other neural systems, particularly the PNS, in co-maintaining bone homeostasis or driving disease progression within the bone microenvironment remains poorly understood. Furthermore, the indirect effect of the SNS on bone-related pathologies via the immune system remains unclear. Specifically, how SNS signaling modulates local immune responses to affect bone metabolism and disease trajectory, as well as the precise “neuro-immune-bone” crosstalk network, warrants systematic investigation.

Future research should prioritize rigorously designed large-scale clinical studies to evaluate the efficacy and safety of SNS-targeting drugs for specific bone diseases while accelerating the preclinical development and translation of novel targeted therapeutics. Crucially, the BBA operates bidirectionally. While this review primarily focuses on descending SNS regulatory mechanisms, future work must explore bone-to-brain feedback loops. The key remaining questions include how bone-derived factors cross the BBB to modulate CNS function and the role of such feedback in regulating neural plasticity and cognition. These insights could identify new therapeutic targets and combinatorial strategies for treating neuropsychiatric comorbidities associated with bone disorders.^156^

In neuro-bone tissue engineering, bone-mimetic hierarchical structures can be recapitulated using layer-by-layer biomimetic techniques^157^ and complemented using smart materials capable of regulating bone-derived signals and neural regeneration.^158^ Artificial intelligence (AI) may further revolutionize this field. Deep-learning algorithms can be used for the high-throughput screening of materials with optimal physicochemical properties (e.g., elastic modulus and hydrophilicity/hydrophobicity) and biological effects (e.g., neurotrophic factor release and neurovascular regeneration efficiency). AI can decode critical nodes within the bone-brain interactome by integrating multi-omics data, such as genomic, proteomic, and imaging analyses, ultimately enabling innovative solutions for personalized precision medicine.^158,159^

## Conclusion

This review has elucidated the emerging concept of the BBA, revealing the intricate bidirectional interplay between the SNS and skeletal system and its profound implications for orthopedic pathologies. Research demonstrates how sympathetic signaling dynamically regulates bone metabolism, inflammatory cascades, osteogenic differentiation, and remodeling processes through neurotransmitter-mediated skeletal homeostasis modulation, which contributes to the pathogenesis of osteoporosis, arthritis, and IVDD. By establishing mechanistic links between sympathetic dysregulation and bone pathophysiology, we propose that the therapeutic targeting of sympathetic pathways and neuroregulatory technologies may pioneer novel paradigms for treating bone-related disorders. However, the full realization of the translational potential of this approach requires further integration of clinical data using advanced technologies. These advances will bridge neurobiology and orthopedics, enabling precision interventions to address the increasing prevalence of skeletal diseases in aging populations.
